# Genomic Analysis of the Endophytic *Stenotrophomonas* Strain 169 Reveals Features Related to Plant-Growth Promotion and Stress Tolerance

**DOI:** 10.3389/fmicb.2021.687463

**Published:** 2021-06-16

**Authors:** Kristina Ulrich, Michael Kube, Regina Becker, Volker Schneck, Andreas Ulrich

**Affiliations:** ^1^Johann Heinrich von Thünen Institute, Institute of Forest Genetics, Waldsieversdorf, Germany; ^2^University of Hohenheim, Stuttgart, Germany; ^3^Leibniz Center for Agricultural Landscape Research (ZALF), Müncheberg, Germany

**Keywords:** plant growth promotion, genome mining, endophytic bacteria, plant-microbe interaction, auxin, phylogenomics

## Abstract

Plant-associated *Stenotrophomonas* isolates have great potential for plant growth promotion, especially under stress conditions, due to their ability to promote tolerance to abiotic stresses such as salinity or drought. The endophytic strain *Stenotrophomonas* sp. 169, isolated from a field-grown poplar, increased the growth of inoculated *in vitro* plants, with a particular effect on root development, and was able to stimulate the rooting of poplar cuttings in the greenhouse. The strain produced high amounts of the plant growth-stimulating hormone auxin under *in vitro* conditions. The comparison of the 16S rRNA gene sequences and the phylogenetic analysis of the core genomes showed a close relationship to *Stenotrophomonas chelatiphaga* and a clear separation from *Stenotrophomonas maltophilia*. Whole genome sequence analysis revealed functional genes potentially associated with attachment and plant colonization, growth promotion, and stress protection. In detail, an extensive set of genes for twitching motility, chemotaxis, flagella biosynthesis, and the ability to form biofilms, which are connected with host plant colonization, could be identified in the genome of strain 169. The production of indole-3-acetic acid and the presence of genes for auxin biosynthesis pathways and the spermidine pathway could explain the ability to promote plant growth. Furthermore, the genome contained genes encoding for features related to the production of different osmoprotective molecules and enzymes mediating the regulation of stress tolerance and the ability of bacteria to quickly adapt to changing environments. Overall, the results of physiological tests and genome analysis demonstrated the capability of endophytic strain 169 to promote plant growth. In contrast to related species, strain 169 can be considered non-pathogenic and suitable for biotechnology applications.

## Introduction

Plant-associated microorganisms are gaining increasing recognition in terms of their importance for plant growth and health. Among them, bacteria of the genus *Stenotrophomonas* are of increasing biotechnological interest due to their potential as effective bioinoculants for plant growth stimulation and for control of a wide range of plant pathogens (Singh and Jha, 2017; An and Berg, 2018; Alexander et al., 2019).

The type species *Stenotrophomonas maltophilia* is a multidrug resistant ubiquitous opportunistic pathogen of environmental, mainly plant-associated, origin, and is commonly found in a wide range of environmental niches (Palleroni and Bradbury, 1993; Ryan et al., 2009; Berg and Martinez, 2015; Gopi et al., 2020). It has also been described as a plant growth-promoting endophyte with beneficial effects due to its production of phytohormones and spermidine, phosphate solubilization, the potential to synthesize siderophores (Singh and Jha, 2017; Abdelaziz et al., 2018; Alexander et al., 2019; Wozniak et al., 2019; Aeron et al., 2020; Alijani et al., 2020) and its capability for bioprotection against fungal and bacterial pathogens (Narayanasamy, 2013; Kumar and Audipud, 2015; Elhalag et al., 2016; Rania et al., 2016). Additionally, some other abilities, including anti-quorum sensing and anti-biofilm bioactivities (Singh et al., 2013; Achari and Ramesh, 2015), as well as the protection against biotic and abiotic stress (Singh and Jha, 2017; Alexander et al., 2019), were demonstrated for this species. The difficulty in distinguishing beneficial from harmful *S. maltophilia* strains compromises the possibility of applications in environmental biotechnology (Berg and Martinez, 2015). However, it was possible to establish a new, clearly plant-associated species, *Stenotrophomonas rhizophila* (Wolf et al., 2002), that could be clearly differentiated from *S. maltophilia* and provides a harmless alternative for biocontrol without human health risks (Berg and Martinez, 2015; Pinski et al., 2020). *S. rhizophila* is a model bacterium for salt-tolerant plant growth-promoting rhizobacteria with an endophytic lifestyle and antifungal properties (Ryan et al., 2009; Alavi et al., 2013b; Egamberdieva et al., 2016). In addition to the production of the plant growth hormone auxin (Suckstorff and Berg, 2003), the production of siderophores (Guerrieri et al., 2020), antifungal volatiles (Kai et al., 2007), spermidine and osmoprotectants (Pinski et al., 2020) has been demonstrated. Schmidt et al. (2012) suggested that *S. rhizophila* may have an indirect plant growth-promoting effect by influencing the microbial community. Two other plant-associated species of the genus *Stenotrophomonas* that are beneficial to plant growth are *Stenotrophomonas chelatiphaga* and *Stenotrophomonas pavanii*. *S. chelatiphaga* (Kaparullina et al., 2009) was described as a siderophore-producing rhizobacterium with plant growth-promoting properties (Ghavami et al., 2017). *S. pavanii* was isolated from Brazilian sugar cane and was found to be able to fix nitrogen based on the identification of *nifH* and acetylene reduction (Ramos et al., 2011). Diazotrophic, siderophore-producing isolates of this species could also be detected in the rhizosphere of tomato plants (Guerrieri et al., 2020).

In this study, an endophytic *Stenotrophomonas* strain with plant growth-promoting properties isolated from the aerial parts of poplar was characterized and compared with other plant-associated *Stenotrophomonas* strains. The genome was investigated to reveal features explaining the beneficial effects for plant development and involved in stress tolerance.

## Materials and Methods

### Isolation and Screening of Endophytic Bacteria From Poplar With Plant Growth-Promoting Potential

The isolation of endophytic bacteria from different field-grown poplar clones was described by Ulrich et al. (2008b). Out of the 513 isolates taxonomically classified at the genus level, 30 representative strains were screened for their plant growth-promoting potential on *in vitro* explants of the hybrid poplar clone 741♀ [*P. alba* × (*P. davidiana* + *P. simonii*) × *P. tomentosa*] (Tian et al., 2000). Explants free from culturable bacteria were obtained from regenerated shoot meristems (Ulrich et al., 2008a). For inoculation, explants were dipped in an overnight TSB (Sigma-Aldrich, Taufkirchen, Germany) culture of the respective bacterial isolate (adjusted to 3 × 10^7^ cells mL^–1^) for 2 s and planted in 50 mL glass tubes containing 10 mL BEMB200 *in vitro* medium (Ewald and Süss, 1993). The control cuttings were given a quick dip in TSB. Eight plants were inoculated for each bacterial isolate. Root and sprout length and the number of main roots were recorded 4 weeks after inoculation.

### Test of *Stenotrophomonas* sp. 169 on Human Pathogenicity

The possible pathogenic potential of strain 169 was investigated by different procedures. A very simple criterion in differentiating between pathogenic and non-pathogenic *Stenotrophomonas* strains is the capability of growing at the human body temperature (Alavi et al., 2014; Berg and Martinez, 2015). Furthermore, extracellular DNase activity is known to be involved in the dissemination of infecting bacteria and Buchanan et al. (2006) demonstrated a role for extracellular DNases in host immune evasion by the degradation of neutrophil extracellular traps (NETs). Third, hemolytic activity is a possible indication of pathogenicity. Accordingly, the strain 169 was incubated on R2A and nutrient agar (Oxoid—Thermo Fisher Scientific, Germany) at 37∘C for 3 days. The strain was also cultivated and monitored on DNase agar and Columbia blood agar (Oxoid—Thermo Fisher Scientific, Germany) as described by the manufacturer.

### Analysis of the Plant Growth Promotion Activity

The effect of strain 169 was validated in a second, more extended *in vitro* test. Forty explants each were used for inoculation and the control. The plants were grown in 100 mL Erlenmeyer flasks as described above. Root and sprout length, number of main roots, and root and sprout weight were recorded 5 weeks after inoculation.

Strain 169 was also tested for the stimulation of rooting of poplar cuttings [clone Geneva; *P. maximowiczii* × (*P. laurifolia* × *P. nigra*)] under greenhouse conditions. Fifty plants each for the inoculated variant and the control were grown in containers (60 × 22 × 12 cm) filled with nutrient-free quartz sand. Before planting, the lower portion of the cuttings (2–3 cm) was inoculated for 2–6 h with an overnight culture of *Stenotrophomonas* sp. 169 grown in R2 broth (adjusted to 1 × 10^8^ cells mL^–1^) or pure growth medium as a control. Ten weeks after inoculation, the total root weight was measured.

The significance of differences between the inoculation variant and the untreated control was analyzed by applying the Wilcoxon rank sum test with continuity correction. Statistical analysis was carried out in R version 3.5.3 (R Core Team, 2019).

### Auxin Quantification

The production of auxins was determined using a microplate method as described by Anguiano-Cabello et al. (2017). For the colorimetric assay, strain 169 was precultivated in R2A growth medium and incubated according to the procedure of Suckstorff and Berg (2003). For calibration curves, indole-3-acetic acid at concentrations of 80–0.078 μg/mL was applied. The test is based on the Salkowski’s reagent and is not specific for only IAA, but the indole derivates indole-pyruvate and indole-acetamide could also form complex with the reagent. However, the principal auxin detected is IAA (Anguiano-Cabello et al., 2017).

### Genome Sequencing

*Stenotrophomonas* sp. 169 was cultured in R2A medium for 2 days at 25∘C. The cells were washed three times with 0.3% NaCl to remove exopolysaccharides, and genomic DNA was subsequently extracted by the Genomic-Tip 20 Kit (Qiagen) according to the manufacturer’s instructions. The purity, fragment size, and quantity of the isolated DNA were assessed on a 1% agarose gel and by using a NanoDrop ND-1000 spectrophotometer (Thermo Scientific). DNA was sequenced using the Pacific Biosciences (PacBio) RS II sequencing platform at GATC Biotech AG (Konstanz, Germany). From the genomic DNA, a 20 kb insert size library was prepared and sequenced using 2 SMRT cells and a 240 min movie time. Sequence reads were *de novo* assembled using the PacBio hierarchical genome assembly process (HGAP3). The assembly resulted in one contig with an average genome coverage of 195x. The genome sequence was circularized with Circlator ver. 1.5.5 and deposited in the GenBank database under accession no. CP061204.

## Bioinformatic Analysis

The genome was annotated with Rapid Annotation using Subsystem Technology (RAST) version 2.0 (Aziz et al., 2008; Overbeek et al., 2014). For comparison of genomes, *S. maltophilia* R551-3 (CP001111), *S. chelatiphaga* DSM 21508^T^ (LDJK00000000), and *S. rhizophila* DSM 14405^T^ (CP007597) were also annotated on the RAST platform to avoid bias derived from using different annotation systems. The calculation of orthologous genes was based on the predicted coding sequences with an identity of more than 70% at the amino acid level. A Venn diagram was generated using the R package VennDiagram (Chen, 2018). Functional and pathway analyses were also performed using the BlastKOALA web tool of the KEGG database (Kanehisa et al., 2016).

For the phylogenetic analysis, an alignment of the 16S rRNA genes from closely related strains and species was generated using the ClustalW algorithm (Larkin et al., 2007) with MEGA X (Kumar et al., 2018). The alignment length was 1470 nt for the nearly total 16S rRNA gene. The tree was constructed using the maximum-likelihood method based on evolutionary distances of the HKY model (+ G + I). The phylogenomic analysis based on core genome phylogeny (Parks et al., 2018) was performed as described by Behrendt et al. (2021). The analysis of 120 bacterial core marker genes resulted in a concatenated amino acid sequence alignment which was used to construct a maximum-likelihood tree (LG substitution model with F + G + I) with MEGA.

The average nucleotide identity (ANI) values were calculated using the OrthoANIu algorithm (Yoon et al., 2017). The average amino acid identity (AAI) was calculated based on the RAST sequence-based comparison by the AAIr calculator.

## Results and Discussion

### Screening of the Endophytic Isolates

**FIGURE 1:**
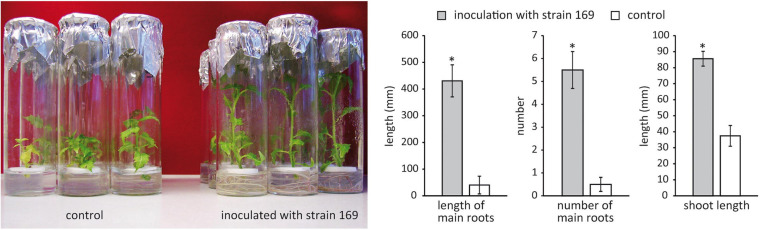
Effect of *Stenotrophomonas* strain 169 on the performance of the *in vitro* poplar clone 741 in comparison to that of the uninoculated control 4 weeks after inoculation. Differences in root and shoot length as well as the number of roots are shown in the bar charts. Stars indicate significant differences (*p* < 0.001, Wilcoxon rank sum test), vertical error bars display standard error.

Thirty endophytic bacterial strains isolated from field-grown poplar (Ulrich et al., 2008b) were screened for plant growth-promoting activity on *in vitro* poplar plants. Isolates belonging to the genera *Pseudomonas*, *Stenotrophomonas*, *Rhizobium*, *Paenibacillus*, and *Microbacterium*, which are known for their putative plant growth-promoting potential, were chosen for the first screening. The effect of *Stenotrophomonas* strain 169 was particularly striking, showing a significant increase in both root and shoot length of the inoculated *in vitro* plants compared to the control (*p* < 0.001) (Figure 1). The strain was originally isolated from surface-disinfected branch sections, which were obtained from 2-year-old shoots of the field-grown hybrid poplar clone 741 at the clonal archive in Waldsieversdorf. The endophytic strain 169 was subjected to a detailed analysis.

### Plant Growth-Promoting Characteristics of *Stenotrophomonas* Strain 169

**FIGURE 2:**
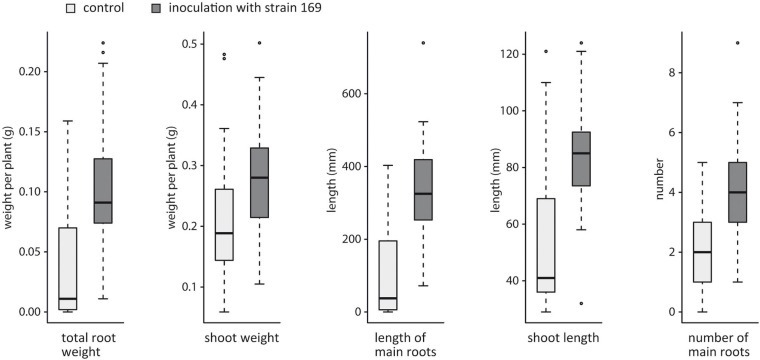
Effects of strain 169 on root and shoot development of the hybrid poplar clone 741 *in vitro*. All measured parameters were significantly increased in the inoculated plants (*p* < 0.0001, Wilcoxon rank sum test).

For the validation of the plant growth-promoting characteristics of strain 169, additional growth parameters were tested in a larger number of individual plants. Five weeks after inoculation, significant effects on shoot and root growth were observed. The root weight of the inoculated plants was three times higher than that of the untreated plants, suggesting that the inoculation has a particular effect on root development (*p* = 7.3e-09) (Figure 2). Accordingly, the length and number of main roots were significantly higher in the inoculated plants than in the control plants (*p* = 4.1e–11, *p* = 1.1e-08). The values for the shoot length and shoot weight were also significantly increased by the influence of this bacterium (*p* = 3.1e-07, *p* = 8.6e-05) (Figure 2), but overall, the effect of the *Stenotrophomonas* strain was more pronounced on root growth.

Under greenhouse conditions, strain 169 was used to stimulate rooting of poplar cuttings. As a result, the inoculated cuttings showed significantly increased root development after 10 weeks, with 0.39 ± 0.024 vs. 0.22 ± 0.017 g FW roots (mean ± SE, *p* = 3.4e–05).

### Genome Sequence Data of *Stenotrophomonas* sp. 169

Sequencing and *de novo* assembly of strain 169 resulted in a complete genome sequence with one contig. Finally, a sequence of 4,157,065 bp for the circular genome was generated. The genome of strain 169 contained 3,703 predicted coding sequences. Genome characteristics were summarized in comparison to the genomes of the plant-associated isolates *S. maltophilia* R551-3, an endophytic bacterium from *Populus* (Taghavi et al., 2009), and *S. rhizophila* DSM 14405^T^, derived from the rhizosphere of rape (Wolf et al., 2002; Table 1). Additionally, the isolate *S. chelatiphaga* DSM 21508^T^, which is most closely related to *Stenotrophomonas* sp. 169 according to the phylogenetic analysis, was included in the comparison (see below). General genome characteristics, e.g., the GC content or the rRNA gene numbers, were in comparable ranges and common for the genus *Stenotrophomonas*.

**TABLE 1:** Summary of the genomic characteristics of *Stenotrophomonas* sp. 169 in comparison to *S. maltophilia* R551-3, *S. rhizophila* DSM 14405^T^, and *S. chelatiphaga* DSM 21508^T^.

	***Stenotrophomonas* sp. 169**	***S. maltophilia* R551-3**	***S. chelatiphaga* DSM 21508^T^**	***S. rhizophila* DSM 14405^T^**
Contigs	1	1	148	1
Number of bases (bp)	4,157,065	4,573,969	3,967,734	4,648,976
G + C content (%)	65.6	66.3	66.8	67.3
Number of predicted coding sequences	3703	4192	3590	4151
Coding base count (%)	90.69	91.47	90.18	90.31
rRNA (16S, total)	3 (10)	4 (13)	1 (5)	3 (12)
tRNA	66	73	62	67

**FIGURE 3:**
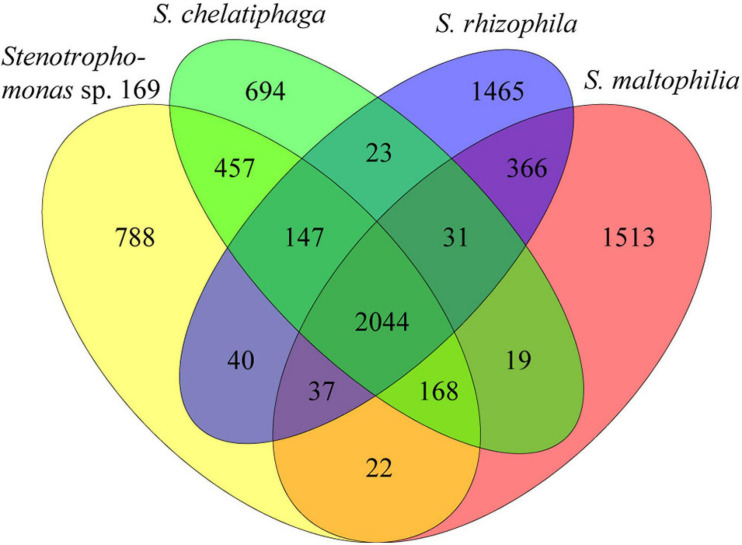
Comparison of orthologous genes between the genomes of *Stenotrophomonas* sp. 169, *S. chelatiphaga* DSM 21508^T^, *S. rhizophila* DSM 14405^T^, and *S. maltophilia* R551-3. The number of shared and genome-specific genes is shown.

The genomes of these four plant-associated *Stenotrophomonas* strains shared 2,044 orthologous protein-coding genes (Figure 3), which accounted for as many as 52.3% of all protein-coding genes. A total of 2,816 genes of strain 169 were orthologous in *S. chelatiphaga* (76.0%), whereas strain 169 shared only 61.3% of its genes with *S. maltophilia* R551-3. The presence of 788 genes (21.3%) found only in the genome of strain 169 indicated its uniqueness.

Genes encoding for proteins that are associated with the pathogenicity of the nosocomial *S. maltophilia* isolate K279a, such as the O-antigen biosynthesis aminotransferase (B2FLT1), a Hep Hag family adhesin (B2FJL8) and the cell surface hemagglutinin gene (B2FLL9) (Crossman et al., 2008; Puopolo et al., 2016), were absent in the genome of strain 169. In this context, three phenotypic criteria were used to distinct strain 169 from harmful *Stenotrophomonas* strains. As a result, strain 169 was unable to grow at 37∘C, did not show extracellular DNase activity and was hemolytic-negative. Together with the clear phylogenetic discrimination from *S. maltophilia* and the absence of genes related to the pathogenicity, it is strongly suggested that strain 169 can be considered non-pathogenic (Alavi et al., 2014; Berg and Martinez, 2015).

### Phylogenetic Position of *Stenotrophomonas* sp. 169

Phylogenetic analysis based on 16S rRNA sequences showed a close relationship of strain 169 to *Stenotrophomonas* strain Fa6 (AY131216), which was isolated as an epiphytical colonizer of strawberry plants by Krimm et al. (2005) (Supplementary Figure 1). Both strains formed a branch with *S. chelatiphaga* supported by a high bootstrap value. Again, this branch clustered with the *S. tumulicola*, however, without significant bootstrap support.

**FIGURE 4:**
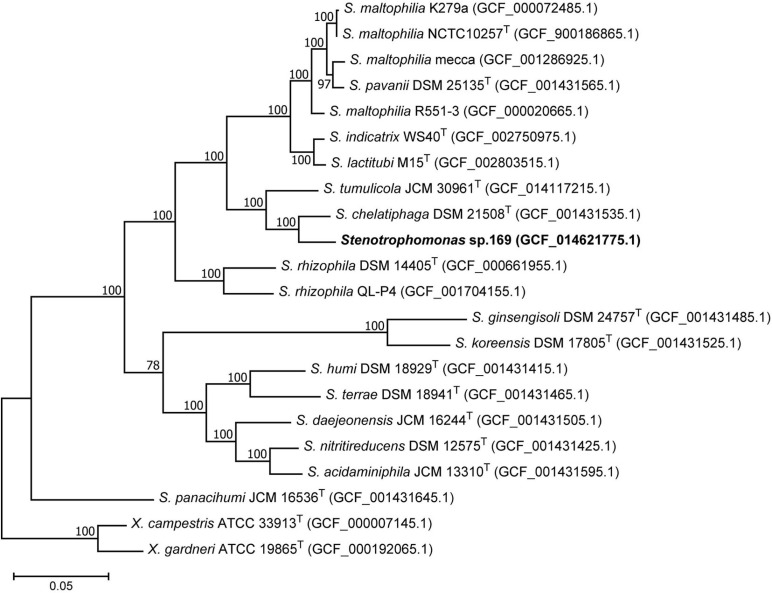
Phylogenomic tree showing the position of *Stenotrophomonas* sp. 169 among type strains and other reference strains of closely related *Stenotrophomonas* species. *Xanthomonas campestris* and *Xanthomonas gardneri* were used as outgroup. The maximum-likelihood tree is based on concatenated 120 core marker proteins. Numbers at branch nodes refer to bootstrap values > 70%. Bar: amino acid substitutions per position. Assembly accession numbers are indicated in brackets.

Due to the high sequence similarity of the 16S rRNA gene within the genus *Stenotrophomonas*, the analysis was completed by core genome phylogeny. The phylogenomic tree again showed a close relationship between strain 169 and *S. chelatiphaga* (Figure 4). Both strains clustered together with *S. tumulicola* supported by a high bootstrap value. This branch was clearly separated from the cluster containing the *S. maltophilia* strains. The next related cluster comprised the two plant-associated *S. rhizophila* strains.

Despite the close relationship shown in both trees, strain 169 could not be classified as *S. chelatiphaga*. The average nucleotide identity (ANI) of the genome sequences of *Stenotrophomonas* sp. 169 and the type strain of *S. chelatiphaga* was 85.5% and accordingly below the value recommended as the cutoff for species demarcation (95%; Lee et al., 2016). Similarly, the AAI value supported a distinction from *S. chelatiphaga* (87.9%).

### Analysis of the Genome Sequence for Plant Growth-Promoting Features

To understand the plant growth-promoting activity of strain 169, a functional analysis of the annotated genome was performed. The *Stenotrophomonas* sp. 169 genome contained a large number of metabolic genes involved in carbohydrate and amino acid metabolism, as well as genes essential for environmental information processing. However, the analyses in this study were focused on the identification of genes for plant growth-promoting compounds such as polyamines and auxins, factors that enhance colonization, stress protection, and other functions concerning bacteria-plant interactions.

#### Production of Polyamines

Polyamines, including putrescine, spermine, spermidine, and cadaverine, play important physiological roles and may be involved in plant-protection and growth promotion (Couee et al., 2004; Al-Whaibi et al., 2012; Xie et al., 2014). In different plants, they were described to correlate with lateral root development and may be implicated in the establishment of biotic interactions between roots and rhizospheric microorganisms (Jang et al., 2002; Niemi et al., 2002; Couee et al., 2004; Hummel et al., 2004; Xie et al., 2014). Additionally, polyamines could protect plants against various environmental stresses, including oxidative, acidic, and osmotic stresses (Kasukabe et al., 2004; Yamaguchi et al., 2007). Thus, using a transcriptomic approach of oilseed rape, spermidine produced by the endophytic isolate *S. rhizophila* DSM 14405^T^ could be identified as a key factor in stress protection in roots in addition to its plant growth-promoting activity (Alavi et al., 2013b). Moreover, spermidine affected biofilm formation and colonization processes via multiple pathways that involved both transport and signaling networks (McGinnis et al., 2009).

**FIGURE 5:**
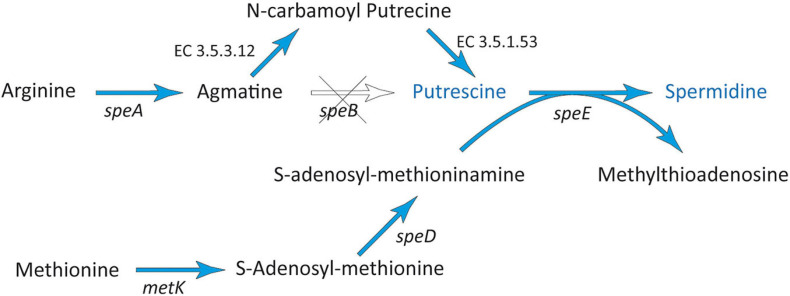
Spermidine biosynthesis pathway found in *Stenotrophomonas* sp. 169. The scheme is based on Jelsbak et al. (2014) and Xie et al. (2014). Blue arrows indicate genes found in the genome of strain 169.

The synthesis of polyamines usually starts with the decarboxylation of the amino acids ornithine or arginine. In the genome of strain 169, all genes coding for enzymes essential for the most prevalent route, starting with the decarboxylation of arginine by arginine decarboxylase (SpeA) (Burrell et al., 2010), could be detected (Figure 5 and Supplementary Table 1). The intermediate agmatine is further converted to putrescine via *N*-carbamoyl-putrescine using agmatine deiminase (EC 3.5.3.12) and *N*-carbamoyl-putrescine amidase (EC 3.5.1.53) (Michael, 2016; Vilas et al., 2018). Finally, strain 169 contained genes for the conversion of putrescine to spermidine (*speE*). For this conversion, S-adenosyl-methioninamine is needed, which was produced from methionine via S-adenosyl-L-methionine by the enzymes MetK and SpeD (Jelsbak et al., 2014; Xie et al., 2014). Additionally, a gene encoding CadA was identified mediating the decarboxylation of L-lysine to produce the polyamine cadaverine, which may also be involved in the promotion of root growth and stress protection (Supplementary Table 1). One may speculate, if the toxic diamine cadaverine could also have a protective effect against infection by phytopathogens.

The potential ability of strain 169 to synthesize putrescine, spermidine, and cadaverine could be an explanation for the promotion of shoot and especially root growth of inoculated *in vitro* poplar plants, as well as the increased root development of poplar cuttings under greenhouse conditions. Because polyamines play a significant role in various bacteria-plant interactions, plant growth, and developmental processes, as well as in stress protection, they are considered a new kind of plant biostimulant (Chen et al., 2019).

#### Biosynthesis of IAA

Endophytic bacteria may enhance plant growth through the synthesis of the hormone indole-3-acetic acid (IAA), which is the most common auxin and is widespread among plant-associated bacteria. In addition to directly promoting plant growth and development, microbial IAA has also been reported to have a major impact on microorganism-plant interactions and plant immunity and also as a signaling molecule (Spaepen et al., 2007; Spaepen and Vanderleyden, 2011; Duca et al., 2014).

The production of IAA-like compounds by *Stenotrophomonas* sp. 169 was shown in culture medium containing tryptophan. The concentration of IAA achieved 47.8 ± 2.98 μg/mL (mean ± SD), which was comparable to other *Stenotrophomonas* strains with a high producing ability. The endophytic *S. maltophilia* strain BE25 from banana produced 39 μg/mL IAA (Ambawade and Pathade, 2015) and the *S. maltophilia* strain ES2 from common horsetail 19.2 μg/mL auxin (Wozniak et al., 2019). Within the species *S. rhizophila*, auxin-producing strains isolated from the rhizosphere of different plants ranged in IAA concentration from 33 μg/mL for the strain UC4098 (Guerrieri et al., 2020) to 78 μg/mL for the strain AJK-9 (Majeed et al., 2015). In general, the amount of auxin production seems to vary strongly between the different strains of *Stenotrophomonas*, even when they belong to the same species (Wozniak et al., 2019; Guerrieri et al., 2020). The results from Suckstorff and Berg (2003) indicated that clinical isolates and aquatic strains of *Stenotrophomonas* produced significantly lower amounts of IAA than plant-associated strains.

Various studies have demonstrated that IAA primarily increases the size and distribution of roots and the number of root hairs, resulting in better nutrient uptake from the soil (Datta and Basu, 2000; Persello-Cartieaux et al., 2003; Mohite, 2013; Gumiere et al., 2014). These results were consistent with the strong root growth of the *in vitro* poplar plants after inoculation with strain 169 and the positive influence of the strain on the rooting of cuttings. However, it should be considered that beside other factors there could also be a combined effect resulting from the action of polyamines and auxin.

All genes necessary for the synthesis of tryptophan, the starting point of IAA synthesis, were annotated in the genome of strain 169 (Supplementary Table 1). Tryptophan biosynthesis usually starts from chorismate, which is transformed to anthranilate by anthranilate synthase (TrpEG). Anthranilate is converted to indole-3-glycerolphosphate in three steps by anthranilate phosphoribosyl transferase (TrpD), phosphoribosylanthranilate isomerase (TrpF) and indole-3-glycerol phosphate synthase (TrpC). This compound can be transformed by tryptophan synthase (TrpAB) directly to tryptophan or enable the generation of indole as an intermediate.

**FIGURE 6:**
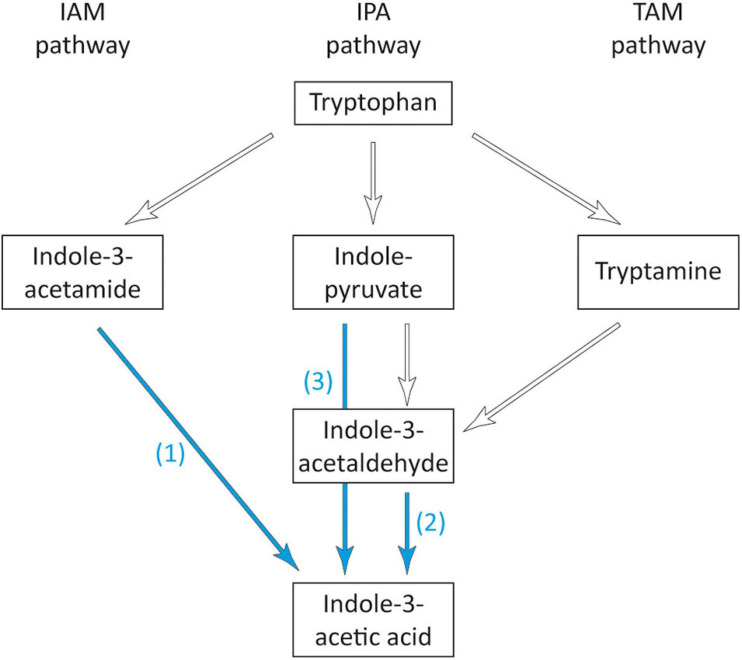
Overview of the bacterial IAA biosynthetic pathways adapted from Spaepen and Vanderleyden (2011). Blue arrows indicate the presence of enzymes detected in *Stenotrophomonas* sp. 169: (1) amidase AmiE, EC 3.5.1.4, (2) aldehyde dehydrogenase, EC 1.2.1.3, (3) indole-pyruvate ferredoxin oxidoreductase, EC 1.2.7.8.

Starting from tryptophan, at least five different pathways have been described for the synthesis of auxin (Spaepen and Vanderleyden, 2011), three of which, proceeding via indole-3-acetamide (IAM), indole-3-pyruvate (IPA), and tryptamine (TAM) are the most common and best characterized pathways for auxin synthesis in bacteria (Cox et al., 2018; Zhang et al., 2019). In the gene inventory of strain 169, three genes encoding for proteins associated with two of these pathways were found (Figure 6 and Supplementary Table 1). For the IAM pathway, the amidase gene *amiE* was detected. The encoded enzyme is able to catalyze the second step from indole-3-acetamide to IAA, similar to the IAM hydrolase IaaH. However, the gene for the enzyme catalyzing the initial conversion from tryptophan to IAM was missing from the genome of strain 169. For the IPA pathway, the gene for the aldehyde dehydrogenase (EC 1.2.1.3), which converts indole-3-acetaldehyde to IAA, could be identified in strain 169. However, the first and second steps from tryptophan via IPA to indole-3-acetaldehyde were not found in the genome. Alternatively, the direct conversion from IPA to IAA by indole-pyruvate ferredoxin oxidoreductase (EC 1.2.7.8) was recently suggested by Imada et al. (2017) and Tullio et al. (2019). This gene was also present in strain 169. Thus, for both pathways, the genes responsible for the initial conversion of tryptophan could not be found. Our results were in correspondence with studies about the IAA-producing strain *S. maltophilia* R551-3, for which enzymes for the same steps of IAA production were found, whereas the genes driving the first step of the IAM pathway and the first two steps of the IPA pathway were lacking (Taghavi et al., 2009). This strongly suggested that in addition to these three described pathways, further enzymes were involved in the initial conversion of tryptophan.

A pathway in which tryptophan is directly converted to indole-3-acetaldehyde by a monooxygenase, the tryptophan side-chain oxidase pathway (TSO), has been reported for some plant-associated bacteria (Suzuki et al., 2003; Duca et al., 2014). However, specific genes or enzymes have not been described or characterized thus far.

#### Siderophore Production and Uptake

Iron is an important micronutrient for a number of vital processes in plants, such as respiration and photosynthesis. Siderophores comprise a high-affinity system for the uptake of iron from the environment through chelation. The ability to produce different kinds of siderophores is widely distributed among plant growth-promoting microorganisms (Scavino and Pedraza, 2013; Pahari et al., 2017). In addition to the positive effect on plant growth by increasing Fe availability for the plant, siderophores can also inhibit the proliferation of various phytopathogens by competition for iron (Solanki et al., 2014; Pahari et al., 2017). Genes responsible for the synthesis and uptake of siderophores could be found in the genome of strain 169 (Supplementary Table 1).

#### Phosphorus Solubilization

Phosphate is frequently a limiting factor in plant growth because it is present in soil or in the rhizosphere in an insoluble form that cannot be used by plants. A number of plant-associated bacteria are able to solubilize phosphorus into a form that can be accessible to plants by secreting phosphatases and phytases, which mobilize phosphates from organic compounds (Sharma et al., 2013). Genes encoding acid and alkaline phosphatases and a phytase were found in the genome of strain 169 (Supplementary Table 1). Additionally, genes mediating polyphosphate formation (*ppk*) (Wan et al., 2020) and phosphonoacetate degradation (*phnA*) (Shariati et al., 2017) were present in the genome. Phosphate starvation-inducible glucose dehydrogenase (EC 1.1.5.2) encoded by the *gcd* gene may also be involved in the solubilization of mineral phosphates (Sharma et al., 2005). The genome also contained genes for P transporters in the high-affinity ABC Pst system (*pstSABC*, see below) and the low-affinity P transport system Pit (Harris et al., 2001).

#### Colonization and Biofilm Formation

Endophytic colonization comprises the adhesion of bacterial cells to the plant surface, the entry, the growth, and the distribution of endophytes within the host plant. Many genes involved in biofilm production, adhesion, motility, and determinants of chemotaxis were hypothesized to contribute to plant colonization and the endophytic life style within the host plant (Venieraki et al., 2016; Kandel et al., 2017). The genome of the endophytic strain 169 contained flagellar protein-coding genes, including the *flg* (flagellar basal body) operon, the *fli* cluster and *flhAB* as well as the genes for the stator unit MotAB and the sigma factor FliA. The genes *pilA*, *pilB*, and *pilT* and the genes for the regulators PilZ and PilX were shown to be responsible for the biogenesis and regulation of type IV pili (Dunger et al., 2014). The genes *cheA*, *cheY*, *cheV*, and *cheW* have been reported to belong to the core chemotaxis genes (Fitzgerald et al., 2014; Reuter et al., 2020; Supplementary Table 1).

Genes for the two site-specific recombinases XerC and XerD, which have been linked to competitive colonization of root surfaces and the rhizosphere by Martinez-Granero et al. (2005) could also be identified in strain 169. The *thuA* gene, which was found in *S. rhizophila* strains (Pinski et al., 2020), may increase the ability of the bacteria to colonize the plant, especially during the early stages. Additionally, genes encoding the multidrug efflux pump (*smeDEF*) were found to be present in strain 169 as well (Supplementary Table 1). Notably, the SmeDEF pump, which was originally considered the most important quinolone resistance determinant of microorganisms, was found to participate in endophytic colonization of plant roots, which could be shown by deletion of the gene *smeE*, which resulted in a reduced ability to colonize the plant (García-León et al., 2014).

The ability to synthesize lipopolysaccharides (LPS) and exopolysaccharides (EPS) is strongly connected with biofilm formation capability. Mutations in related genes affected biofilm development and twitching motility in the clinical *S. maltophilia* strain WR-C (Huang et al., 2006). In the plant growth-promoting *S. rhizophila* strain DSM 14405^T^, LPS/EPS-coupled biosynthetic genes were upregulated as a result of salt stress, suggesting a function in response to changing osmotic conditions (Alavi et al., 2013b). Both the gene inventory for nucleotide-sugar and dTDP-L rhamnose biosynthesis (*xanAB* and *rfbABCD*) (Vorhölter et al., 2001; da Silva et al., 2002; Huang et al., 2006; Supplementary Table 1) and the colony morphology characterized by extended mucus formation suggested EPS production for strain 169.

In addition to motility, chemotaxis and the ability to form biofilms, the degradation of plant polymers has been proposed to be necessary for colonization by bacteria (Reinhold-Hurek et al., 2006; Hardoim et al., 2008; Calderan-Rodrigues et al., 2018). Genes coding for different hydrolytic enzymes, such as glycoside hydrolases, which may allow entry into and translocation within the plant, were present in the genome of strain 169.

The endophytic lifestyle requires a broad spectrum of genes involved in the uptake and transport of nutrients from the plant for bacterial growth and reproduction. In the genome of strain 169, genes encoding different transport systems, such as ABC and MFS transporters, were found. Two transport systems that may play central roles in the control and coordination of carbon, exogenous hexosephosphate, and phosphorous metabolism are the sugar-transporting phosphotransferase system (PTS) (Deutscher et al., 2006) and the phosphate-specific ABC transporter complex PstSABC (Västermark and Saier, 2014). In *Stenotrophomonas* sp. 169, genes coding for the PTS sugar uptake system (*ptsPI*, *hprK, EIIMan*) belonging to the fructose family were annotated (Supplementary Table 1). The plant growth-promoting *S. maltophilia* strain R551-3, isolated as an endophytic bacterium from poplar, possessed comparable features with a single gene coding for a PTS from the fructose family (Taghavi et al., 2009).

#### Genes Involved in Regulation and Secretion Systems

In addition to the manifestation of various phenotypic characteristics in gram-negative bacteria, the production of several bioactive compounds has been shown to be regulated by the diffuse signal factor (DSF) system. This system is based on a mechanism by which small signaling molecules such as N-acyl homoserine lactones are used for cellular communication, allowing bacteria to regulate gene expression in a cell density-dependent manner (Barber et al., 1997). In the multidrug-resistant opportunistic pathogen *S. maltophilia* K279a, the DSF system mediates the production of extracellular protease, β-lactam resistance and virulence (Alcaraz et al., 2019). However, various studies demonstrated that the DSF system is also of importance for plant-associated bacteria (Müller et al., 2009; Pinski et al., 2019). DSF-based molecules are involved in the regulation of spermidine synthase and other genes linked to plant growth promotion and plant colonization (Müller et al., 2009; Alavi et al., 2013a; Suppiger et al., 2016). All components of the DSF system encoded by the *rpf* cluster (Huedo et al., 2015) were present in the genome of *Stenotrophomonas* sp. 169 (Supplementary Table 1) as well as in *S. maltophilia* and *S. rhizophila*. For strains of these species, a clear dose-dependent effect on the growth promotion of strawberry plants could be shown (Suckstorff and Berg, 2003).

In the genome sequence of strain 169, genes encoding type I, II, III, and IV secretion systems were detected. Protein secretion systems are involved in a wide range of biotic interactions (Green and Mecsas, 2016). In contrast to strain 169, the endophytic *S. maltophilia* strain RR-10, isolated from rice roots, lacked the type III secretion system (T3SS) (Zhu et al., 2012). The same was found for the bacteremia-associated isolates *S. maltophilia* K279a (Crossman et al., 2008) and *S. rhizophila* DSM 14405^T^ (Alavi et al., 2014). Originally, the T3SS was described as a key pathogenic factor in gram-negative bacteria that enabled the transfer of bacterial effector proteins directly into host cells (Büttner and Bonas, 2006). Investigations on plant-associated bacteria showed that T3SS-secreted proteins were not restricted to pathogenic bacteria, but also played a role in mutualistic rhizobia (Viprey et al., 1998; Marie et al., 2001; Gourion et al., 2015) and other plant growth-promoting bacteria, such as fluorescent pseudomonads (Zamioudis and Pieterse, 2012; Stringlis et al., 2019). In rhizobia, effectors delivered by the T3SS were involved in the suppression of host immunity and determination of host specificity and nodulation (Okazaki et al., 2013; Gourion et al., 2015). Homologs of all nine genes encoding components of the T3SS on the symbiotic plasmid of *Rhizobium* sp. NGR234 (Viprey et al., 1998) were present in the genome of strain 169 (*sctC,J,R,S,T,U,V,Q,N*) (Supplementary Table 1). Recently, Pinski et al. (2019) demonstrated that inactivation of the T3SS in endophytic strains led to decreased colonization capabilities. Thus, it is suggested that the T3SS functions similarly in *Stenotrophomonas* sp. 169.

#### Reaction to Abiotic Stress Conditions

Endophytic bacteria must have the ability to quickly adapt to different habitats and to cope with plant defense mechanisms, such as the production of highly reactive chemical molecules (Taghavi et al., 2010; Mitter et al., 2013). A number of genes, such as glutathione S-transferase, glutathione synthase, alkyl hydroperoxide reductase, catalases, and peroxidases, participate in the detoxification of these reactive electrophilic compounds could be identified in the genome of strain 169 (Supplementary Table 1).

Genome analysis also revealed the presence of genes related to osmoregulation. One of the early events in responses to osmotic stress in bacteria is the controlled uptake and accumulation of potassium, which, along with its counterion glutamate, may be involved in signal transduction for secondary responses in osmoregulation (Booth and Higgins, 1990). Strain 169 possessed genes encoding K^+^ uptake transporters such as the Kdp-ATPase system (*kdpABCDE*) and Kup (*kup*) (Sleator and Hill, 2002) and a potassium efflux system (*kefA, kefB, kefC*) (Booth et al., 1993; Supplementary Table 1). As a secondary reaction to osmotic stress, cells accumulate high levels of low molecular weight water-soluble compounds known as “compatible solutes” or “osmolytes,” such as amino acids, betaines, polyols, or sugars. In the genome of strain 169, three genes (*proABC*) coding for the synthesis of the amino acid proline starting from glutamate could be identified (Zhang et al., 2002). Glycine betaine can be produced via a two-step enzymatic reaction (*betA*, *betB*) starting from choline (Mandon et al., 2003). For the synthesis of the disaccharide trehalose, two different pathways (*otsAB* and *treY*, *treZ*) were found in strain 169 (Supplementary Table 1). The *otsA-otsB* pathway is the most widespread pathway and is found in all prokaryotic organisms that synthesize trehalose from glucose through the trehalose-6-phosphate synthase/phosphatase pathway (Paul et al., 2008). Overproduction of trehalose by plant-associated microorganisms could indirectly increase the tolerance of host plants to abiotic stresses such as drought, high salinity and extreme temperature, resulting in plant growth promotion (Glick, 2012; Khan et al., 2016). *Phaseolus vulgaris* plants inoculated with *Rhizobium etli* overexpressing *otsA* revealed increased drought tolerance as well as increased biomass production (Suárez et al., 2008). In a similar experiment, a trehalose-overexpressing *Azospirillum* strain achieved significant growth promotion in *Zea mays*, in addition to a more pronounced drought stress tolerance (Rodríguez-Salazar et al., 2009). In addition to the production of trehalose, the gene encoding glucosylglycerol-phosphate synthase (*ggpS*) was also found in the genome of strain 169, which is required for the production of the osmoprotective glycoside glucosylglycerol (Hagemann and Erdmann, 1994; Supplementary Table 1). In *Stenotrophomonas*, the ability to synthesize this osmoprotective glycoside in addition to trehalose seems to be strain-dependent. Whereas the type strain of *S. maltophilia* DSM 50170^T^, a clinical isolate, accumulated only trehalose (Roder et al., 2005), five out of nine rhizosphere isolates of this species accumulated glucosylglycerol in combination with trehalose (Mikkat et al., 2000). The plant growth-promoting strain *S. rhizophila* DSM 14405^T^ was also described to synthesize glucosylglycerol as a compatible solute (Wolf et al., 2002; Roder et al., 2005). Using transcriptomic approaches, the production of glucosylglycerol was found to be a remarkable mechanism to protect roots against osmotic stress (Alavi et al., 2013b). The ability of certain *Stenotrophomonas* strains to mediate stress tolerance enables colonization of the rhizosphere and endosphere of plants, especially under extreme conditions. Accordingly, colonization with these strains could result in enhanced plant growth, especially in marginal soil.

## Conclusion

In summary, the endophytic *Stenotrophomonas* strain 169, isolated from aerial parts of poplar, increased the root, and sprout weights of inoculated poplar plants *in vitro* and stimulated rooting of poplar cuttings. Genome mining revealed functional genes which may explain the colonization competence of this strain and its ability to promote plant growth. It further indicated features known to be related with the tolerance of plants to abiotic stresses. Specifically, the presence of genes for the production of siderophores and the ability to mobilize phosphates, as well as the production of the plant growth-promoting substances IAA and polyamines, could be associated with the ability of strain 169 to improve plant growth. Furthermore, several functional genes mediating the production of osmolytes were detected in the genome. The accumulation of osmoprotective molecules, as well as the production of polyamines, could give the bacterium the potential to protect plant roots against abiotic stress, ultimately resulting in plant growth promotion.

## Data Availability Statement

The datasets presented in this study can be found in online repositories. The names of the repository/repositories and accession number(s) can be found below: https://www.ncbi.nlm.nih.gov/genbank/, CP061204.

## Author Contributions

KU performed the experiments and prepared the manuscript. AU and KU conducted the sequencing, *de novo* assembly, and phylogenetic analysis of strain 169. KU, RB, and VS performed the *in vitro* and greenhouse tests. KU, AU, and MK performed the genome analyses and comparisons. All authors listed substantially contributed to and approved the manuscript.

## Conflict of Interest

The authors declare that the research was conducted in the absence of any commercial or financial relationships that could be construed as a potential conflict of interest.

## Supplementary Material

Supplementary Figure 1 Maximum-likelihood tree of the 16S rRNA gene sequences showing the position of Stenotrophomonas sp. 169 among type strains and other reference strains of closely related Stenotrophomonas species. Phylogenetic analysis was performed using the HKY+G+I model. Xanthomonas campestris and Xanthomonas gardneri were used as outgroup. Numbers at branch nodes refer to bootstrap values >70%. Bar: substitutions per nucleotide site. Accession numbers (and for genomic sequences additionally locus tags) are indicated in brackets.

Supplementary Table 1 List of genetic features identified in the genome of *Stenotrophomonas* sp. 169, which are potentially involved in colonization, plant development and stress tolerance.
