# Supplementary material for: Genomic Analysis of the Endophytic Stenotrophomonas Strain 169 Reveals Features Related to Plant-Growth Promotion and Stress Tolerance
Source: Front Microbiol. 2021 Jun 16;12:687463. doi: 10.3389/fmicb.2021.687463 (PMC8245107; doi:10.3389/fmicb.2021.687463)
Supplement: Supplementary Figure 1 — Maximum-likelihood tree of the 16S rRNA gene sequences showing the position of Stenotrophomonas sp. 169 among type strains and other reference strains of closely related Stenotrophomonas species. Phylogenetic analysis was performed using the HKY+G+I model. Xanthomonas campestris and Xanthomonas gardneri were used as outgroup. Numbers at branch nodes refer to bootstrap values >70%. Bar: substitutions per nucleotide site. Accession numbers (and for genomic sequences additionally locus tags) are indicated in brackets. [file Data_Sheet_1.DOCX]

Figure S1: Maximum-likelihood tree of the 16S rRNA gene sequences showing the position of *Stenotrophomonas* sp. 169 among type strains and other reference strains of closely related *Stenotrophomonas* species. Phylogenetic analysis was performed using the HKY+G+I model. *Xanthomonas campestris* and *Xanthomonas gardneri* were used as outgroup. Numbers at branch nodes refer to bootstrap values >70%. Bar: substitutions per nucleotide site. Accession numbers (and for genomic sequences additionally locus tags) are indicated in brackets.


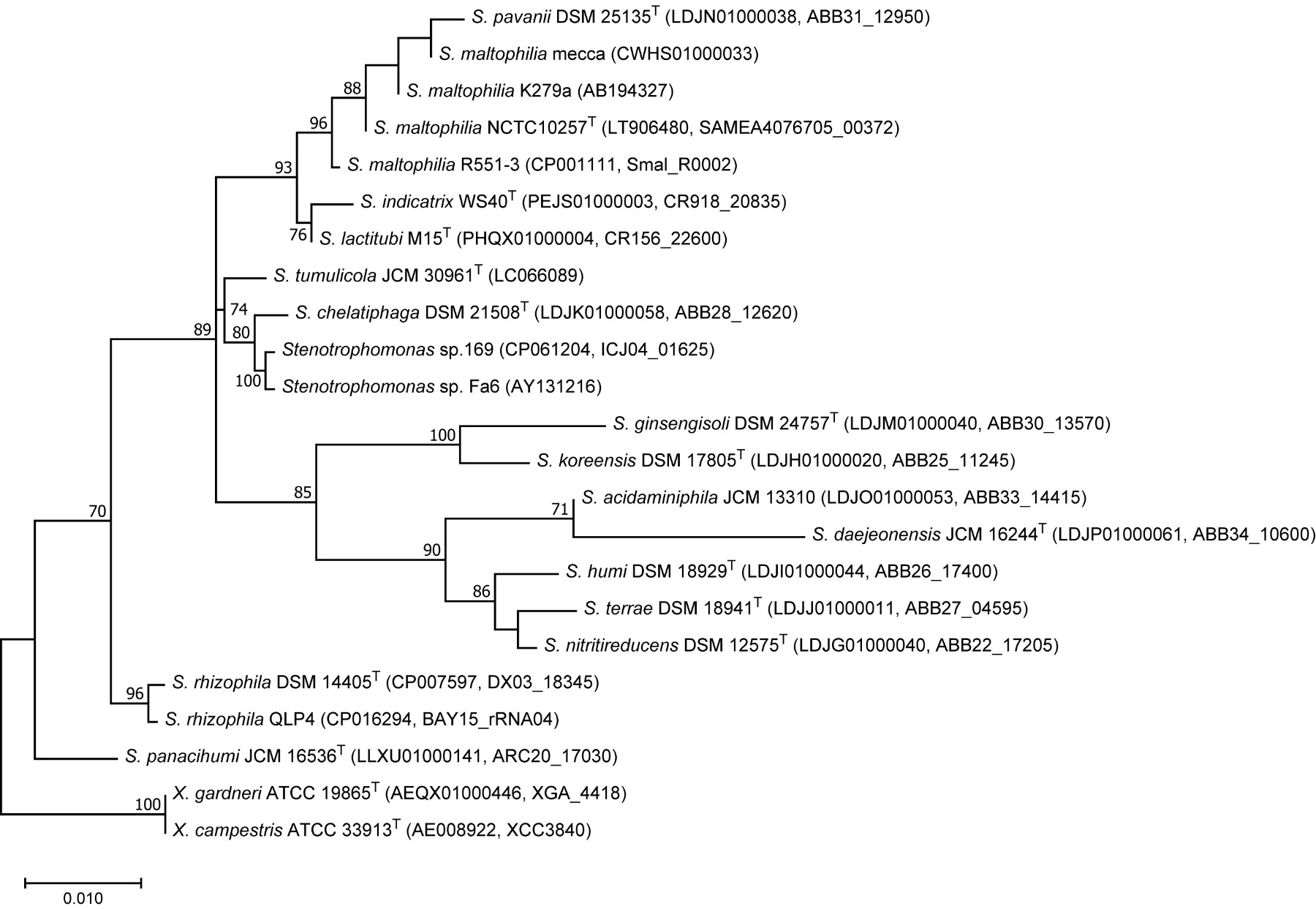


Table S1: List of genetic features identified in the genome of *Stenotrophomonas* sp. 169, which are potentially involved in colonization, plant development and stress tolerance

| Locus tag | Gene | Enzyme code | Product |
| --- | --- | --- | --- |
| ***Production of polyamines*** | | | |
| ICJ04_16790 | *speA* | EC 4.1.1.19 | arginine decarboxylase |
| ICJ04_10845 |  | EC 3.5.3.12 | agmatine deiminase |
| ICJ04_10850 |  | EC 3.5.1.53 | N-carbamoyl putrescine amidase |
| ICJ04_16795 | *speE* | EC 2.5.1.16 | spermidine synthase |
| ICJ04_04565 |  | EC 2.5.1.16 | spermidine synthase |
| ICJ04_16530 | *speD* | EC 4.1.1.50 | S-adenosylmethionine decarboxylase proenzyme |
| ICJ04_03075 | *metK* | EC 2.5.1.6 | methionine adenosyltransferase |
| ICJ04_08470 | *cadA* | EC 4.1.1.18 | cadmium-translocating P-type ATPase |
| ***Biosynthesis of indole-3-acetic acid*** | | | |
| ICJ04_16560 | *trpE* | EC 4.1.3.27 | anthranilate synthase component II |
| ICJ04_16570 | *trpG* | EC 4.1.3.27 | anthranilate synthase component I |
| ICJ04_16555 | *trpD* | EC 2.4.2.18 | anthranilate phosphoribosyltransferase |
| ICJ04_06655 | *trpF* | EC 5.3.1.24 | phosphoribosylanthranilate isomerase |
| ICJ04_16550 | *trpC* | EC 4.1.1.48 | indole-3-glycerol phosphate synthase |
| ICJ04_06670 | *trpA* | EC 4.2.1.20 | tryptophan synthase subunit alpha |
| ICJ04_06665 | *trpB* | EC 4.2.1.20 | tryptophan synthase subunit beta |
| ICJ04_14700 | *amiE* | EC 3.5.1.4 | amidase |
| ICJ04_10640 |  | EC 1.2.1.3 | aldehyde-dehydrogenase |
| ICJ04_00530 |  | EC 1.2.7.8 | indolepyruvate ferredoxin oxidoreductase |
| ***Production and uptake of siderophores*** | | | |
| ICJ04_14515 |  | EC 3.3.2.1 | isochorismatase |
| ICJ04_06625 | *aroC* | EC 4.2.3.5 | chorismate synthase |
| ICJ04_07925 | *aroA* | EC 2.5.1.19 | 3-phosphoshikimate 1-carboxyvinyltransferase |
| ICJ04_15450 | *aroQ* | EC 5.4.99.5 | gamma subclass chorismate mutase |
| ICJ04_07920 | *pheA* | EC 4.2.1.51 | prephenate dehydratase |
| ICJ04_04815  ICJ04_05130 ICJ04_08155  ICJ04_12290 | *tonB* |  | TonB-dependent siderophore receptor |
| ICJ04_06185 |  |  | TonB family protein |
| ICJ04_00045 | *exbB* |  | TonB-system energizer |
| ICJ04_00050 ICJ04_00055  ICJ04_11970 | *exbD* |  | biopolymer transporter |
| ICJ04_11975 | *tolQ* |  | MotA/TolQ/ExbB proton channel family protein |
| ICJ04_03565 | *fhuE* |  | ferric-rhodotorulic acid/ferric-coprogen receptor |
| ICJ04_10270 | *feoA* |  | ferrous iron transporter |
| ICJ04_10265 | *feoB* |  | ferrous iron transporter |
| ICJ04_13480 | *tolA* |  | cell envelope integrity protein |
| ICJ04_15190 | *ahpC* |  | peroxiredoxin |
| ICJ04_13595  ICJ04_13650 |  |  | ferric enterobactin receptor |
| ICJ04_04830 | *fiu* |  | catecholate siderophore receptor |
| ICJ04_12500 | *bfr* |  | bacterioferritin |
| ***Phosphorus solubilization*** | | | |
| ICJ04_06295 | *phoA* | EC 3.1.3.1 | alkaline phosphatase |
| ICJ04_12800 |  | EC 3.1.4.1 | alkaline phosphodiesterase |
| ICJ04_00335 |  | EC 3.1.3.2 | acid phosphatase |
| ICJ04_11260 |  | EC 3.1.3.8 | phytase |
| ICJ04_14555 | *ppx* | EC 3.6.1.11 | exopolyphosphatase |
| ICJ04_03945  ICJ04_11250 | *ppa* | EC 3.6.1.1 | inorganic pyrophosphatase |
| ICJ04_14550 | *ppk* | EC 2.7.4.1 | polyphosphate kinase |
| ICJ04_06020 | *phnA* | EC 3.11.1.2 | phosphonoacetate hydrolase |
| ICJ04_16170 |  |  | TC.GNTP, gluconate:H+symporter, GntP family |
| ICJ04_14025 | *gcd* | EC 1.1.5.2 | phosphate starvation-inducible glucose dehydrogenase |
| ***Colonization and biofilm formation*** | | | |
| ICJ04_09795 | *flgB* |  | flagellar basal body rod protein |
| ICJ04_09810 | *flgE* |  | flagellar hook protein |
| ICJ04_09840 | *flgK* |  | flagellar hook-associated protein |
| ICJ04_09785 | *flgA* |  | flagellar basal body P-ring formation protein |
| ICJ04_09800 | *flgC* |  | flagellar basal body rod protein |
| ICJ04_09820 | *flgG* |  | flagellar basal-body rod protein |
| ICJ04_09815 | *flgF* |  | flagellar basal-body rod protein |
| ICJ04_09860 | *fliD* |  | flagellar filament capping protein |
| ICJ04_09905 | *fliE* |  | flagellar hook-basal body complex protein |
| ICJ04_09910 | *fliF* |  | flagellar M-ring protein |
| ICJ04_09915 | *fliG* |  | flagellar motor switch protein |
| ICJ04_09920 | *fliH* |  | flagellar assembly protein |
| ICJ04_09925 | *fliI* |  | FliI/YscN family ATPase |
| ICJ04_09930 | *fliJ* |  | flagellar export protein |
| ICJ04_09935 | *fliK* |  | flagellar hook-length control protein |
| ICJ04_09940 | *fliL* |  | flagellar basal body-associated FliL famil protein |
| ICJ04_09945 | *fliM* |  | flagellar motor switch protein |
| ICJ04_09960 | *fliP* |  | bacterial flagellar biogenesis protein |
| ICJ04_09965 | *fliQ* |  | flagellar biosynthetic protein |
| ICJ04_09865 | *fliS* |  | flagellar export chaperone |
| ICJ04_09985 | *flhA* |  | flagellar biosynthesis protein |
| ICJ04_09980 | *flhB* |  | flagellar biosynthesis protein |
| ICJ04_02220 | *motA* |  | flagellar motor stator protein |
| ICJ04_02215 | *motB* |  | flagellar motor protein |
| ICJ04_10000 | *fliA* |  | RNA polymerase sigma factor for flagellar operon |
| ICJ04_10015 | *cheA* |  | chemotaxis protein |
| ICJ04_10035 | *cheW* |  | purine-binding chemotaxis protein |
| ICJ04_10090 | *cheR* |  | chemotaxis protein |
| ICJ04_10485 | *cheB* |  | chemotaxis protein |
| ICJ04_10005 | *cheY* |  | chemotaxis response regulator |
| ICJ04_10010 | *cheZ* |  | protein phosphatase |
| ICJ04_09790 | *cheV* |  | chemotaxis protein |
| ICJ04_04560 | *pilA* |  | type IV pilin |
| ICJ04_04570 | *pilB* |  | type IV fimbrial assembly, ATPase |
| ICJ04_13755 | *pilT* |  | twitching motility protein |
| ICJ04_14050 | *pilZ* |  | type IV pilus biogenesis protein |
| ICJ04_12030 | *pilX* |  | type IV fimbrial biogenesis protein |
| ICJ04_13725 | *pilR* |  | sigma-54-dependent Fis family transcriptional regulator |
| ICJ04_13720 | *pilS* |  | sensor histidine kinase |
| ICJ04_04570 | *pilB* |  | type IV-A pilus assembly ATPase |
| ICJ04_12025 | *pilC* |  | pilus assembly protein PilC |
| ICJ04_04550 | *pilD* | EC 3.4.23.43 | prepilin peptidase |
| ICJ04_12020 | *pilE* |  | type IV pilin protein |
| ICJ04_02415 | *pilU* |  | type IV pilus assembly ATPase component |
| ICJ04_03230 | *xerC* |  | tyrosine recombinase |
| ICJ04_02690 | *xerD* |  | site-specific tyrosine recombinase |
| ICJ04_17480 | *thuA* |  | trehalose utilization protein |
| ICJ04_03290 | *smeD* |  | multidrug efflux RND transporter periplasmic adaptor |
| ICJ04_03295 | *smeE* |  | multidrug efflux RND transporter permease |
| ICJ04_03300 | *smeF* |  | efflux transporter outer membrane subunit |
| ICJ04_01550 | *xanA* | EC 5.4.2.2  EC 5.4.2.8 | **phosphohexose mutases, 2 domains (**phosphomannomutase/phosphoglucomutase) |
| ICJ04_02525 | *xanB* | EC 5.3.1.8  EC 2.7.7.13 | mannose-6-phosphate isomerase, mannose-1-phosphate guanylyltransferase |
| ICJ04_02550 | *rfbA* | EC 2.7.7.24 | glucose-1-phosphate thymidylyltransferase |
| ICJ04_02545 | *rfbB* | EC 4.2.1.46 | dTDP-glucose 4,6-dehydratase |
| ICJ04_02555 | *rfbC* | EC 5.1.3.13 | dTDP-4-dehydrorhamnose 3,5-epimerase |
| ICJ04_02560 | *rfbD* | EC 1.1.1.133 | dTDP-4-dehydrorhamnose reductase |
| ICJ04_04625 | *ptsPI* | EC 2.7.3.9 | phosphoenolpyruvate-protein phosphotransferase |
| ICJ04_04645 | *hprK* | EC 2.7.11.- | HPr kinase/phosphorylase |
| ICJ04_04635 | *EIIMan* | EC 2.7.1.191 | **PTS mannose-specific EIIAB component** |
| ICJ04_12360  ICJ04_12365 | *pstS* |  | phosphate ABC transporter substrate-binding protein |
| ICJ04_12375 | *pstA* |  | phosphate ABC transporter permease |
| ICJ04_12380 | *pstB* |  | phosphate ABC transporter ATP-binding protein |
| ICJ04_12370 | *pstC* |  | phosphate ABC transporter permease subunit |
| ICJ04_14545 | *phoR* |  | phosphate regulon sensor histidine kinase |
| ICJ04_12385 | *phoU* |  | phosphate signaling complex protein |
| ICJ04_14540 | *phoB* |  | phosphate regulon transcriptional regulator |
| ICJ04_12355 |  |  | phosphate selective outer membrane porin OprP/OprO |
| ICJ04_12155 | *phoH* |  | phosphate starvation-inducible protein |
| ICJ04_11770 |  |  | TC.PIT inorganic phosphate transporter |
| ***Regulation and secretion systems*** | | | |
| ICJ04_10185 | *rpfG* |  | two-component system response regulator |
| ICJ04_10180 | *rpfC* | EC 2.7.3.- | sensor histidine kinase |
| ICJ04_10175 | *rpfF* |  | DSF synthase |
| ICJ04_17855 | *sctC* |  | T3SS outer membrane ring subunit |
| ICJ04_17745 | *sctJ* |  | T3SS inner membrane ring lipoprotein |
| ICJ04_17815 | *sctR* |  | T3SS inner membrane protein |
| ICJ04_17810 | *sctS* |  | T3SS export apparatus subunit |
| ICJ04_17805 | *sctT* |  | T3SS export apparatus subunit |
| ICJ04_17800 | *sctU* |  | T3SS export apparatus switch protein |
| ICJ04_17845 | *sctV* |  | T3SS export apparatus protein |
| ICJ04_17820 | *sctQ* |  | FliMN family flagellar motor switch protein |
| ICJ04_17835 | *sctN* |  | FliI/YscN family ATPase |
| ICJ04_17850 | *sctW* |  | T3SS gatekeeper subunit |
| ICJ04_17750 | *sctI* |  | T3SS inner rod protein |
| ICJ04_17755 | *sctF* |  | T3SS needle major subunit |
| ICJ04_17790 | *sctE* |  | T3SS translocon subunit |
| ICJ04_11105 | *hrcA* |  | heat-inducible transcriptional repressor |
| ICJ04_17795 | *icrH* |  | T3SS chaperone |
| ICJ04_13390 | *hrpA* | EC 3.6.4.13 | ATP-dependent helicase |
| ICJ04_17225 | *hrpB* | EC 3.6.4.13 | ATP-dependent helicase |
| ICJ04_17780 | *ipaD* |  | T3SS needle tip protein |
| ICJ04_09960 | *fliP* |  | T3SS flagellar biosynthesis inner membrane protein |
| ***Stress reaction*** | | | |
| ICJ04_10980  ICJ04_12115  ICJ04_12145  ICJ04_14665  ICJ04_15925  ICJ04_17130 |  | EC 2.5.1.18 | glutathione S-transferase |
| ICJ04_13335 | *gshB* | EC 6.3.2.3 | glutathione synthase |
| ICJ04_06360 | *gorA* | EC 1.8.1.7 | glutathione-disulfide reductase |
| ICJ04_15190 | *ahpC* | EC 1.11.1.261 | alkyl hydroperoxide reductase |
| ICJ04_15195 | *ahpF* | EC 1.8.1.- | alkyl hydroperoxide reductase subunit F |
| ICJ04_08980  ICJ04_12895  ICJ04_15615 |  | EC 1.11.1.6 | catalase |
| ICJ04_0143 |  | EC 1.11.1.- | peroxidase |
| ICJ04_01575 | *kdpA* | EC 7.2.2.6 | potassium-transporting ATPase subunit |
| ICJ04_01570 | *kdpB* | EC 7.2.2.6 | potassium-transporting ATPase subunit |
| ICJ04_01565 | *kdpC* |  | potassium-transporting ATPase subunit |
| ICJ04_01560 | *kdpD* |  | sensor histidine kinase |
| ICJ04_01555 | *kdpE* |  | response regulator transcription factor |
| ICJ04_13505 | *kup* |  | potassium transporter |
| ICJ04_16745 | *kefA* |  | potassium efflux system |
| ICJ04_10735 | *kefB/C* |  | inner membrane protein |
| ICJ04_13600 |  |  | Kef family K(+) transporter |
| ICJ04_17300 |  |  | cation:proton antiporter, KefB/KefC family |
| ICJ04_06535 | *proA* | EC 1.2.1.41 | glutamate-5-semialdehyde dehydrogenase |
| ICJ04_06525 | *proB* | EC 2.7.2.11 | glutamate 5-kinase |
| ICJ04_13765 | *proC* | EC 1.5.1.2 | pyrroline-5-carboxylate reductase |
| ICJ04_10165 | *betA* | EC 1.1.99.1 | choline dehydrogenase |
| ICJ04_10160 | *betB* | EC 1.2.1.8 | betaine-aldehyde dehydrogenase |
| ICJ04_13665 | *otsA* | EC 2.4.1.15 | alpha,alpha-trehalose-phosphate synthase |
| ICJ04_13655 | *otsB* | EC 3.1.3.12 | trehalose-phosphatase |
| ICJ04_08900 | *treY* | EC 5.4.99.15 | malto-oligosyltrehalose synthase |
| ICJ04_08890 | *treZ* | EC 3.2.1.141 | malto-oligosyltrehalose trehalohydrolase |
| ICJ04_13575 | *ggpS* | EC 2.4.1.213 | glucosylglycerol-phosphate synthase |
| ICJ04_05610 | *katG* | EC 1.11.1.21 | catalase/peroxidase HPI |
